# Fluctuations in the Photoluminescence Excitation Spectra of Individual Semiconductor Nanocrystals

**DOI:** 10.1021/acs.jpclett.4c00516

**Published:** 2024-04-29

**Authors:** Robert C. Keitel, Raphael Brechbühler, Ario Cocina, Felipe V. Antolinez, Stefan A. Meyer, Sander J. W. Vonk, Henar Rojo, Freddy T. Rabouw, David J. Norris

**Affiliations:** † Optical Materials Engineering Laboratory, Department of Mechanical and Process Engineering, ETH Zurich, 8092 Zurich, Switzerland; ‡ Debye Institute for Nanomaterials Science, Utrecht University, 3584 CC Utrecht, The Netherlands

## Abstract

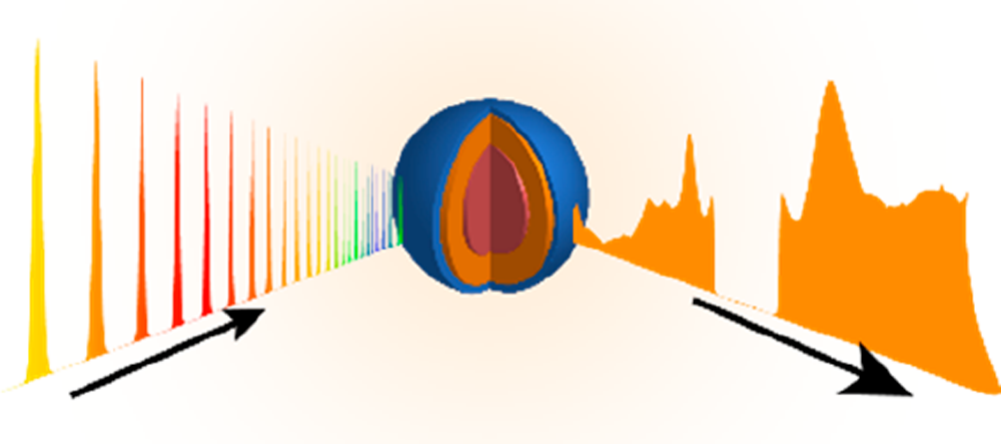

Most single quantum emitters display non-steady emission properties. Models that explain this effect have primarily relied on photoluminescence measurements that reveal variations in intensity, wavelength, and excited-state lifetime. While photoluminescence excitation spectroscopy could provide complementary information, existing experimental methods cannot collect spectra before individual emitters change in intensity (blink) or wavelength (spectrally diffuse). Here, we present an experimental approach that circumvents such issues, allowing the collection of excitation spectra from individual emitters. Using rapid modulation of the excitation wavelength, we collect and classify excitation spectra from individual CdSe/CdS/ZnS core/shell/shell quantum dots. The spectra, along with simultaneous time-correlated single-photon counting, reveal two separate emission-reduction mechanisms caused by charging and trapping, respectively. During bright emission periods, we also observe a correlation between emission red-shifts and the increased oscillator strength of higher excited states. Quantum-mechanical modeling indicates that diffusion of charges in the vicinity of an emitter polarizes the exciton and transfers the oscillator strength to higher-energy transitions.

The optical properties of semiconductor nanocrystals (or colloidal quantum dots, cQDs) can be tuned by controlling their size and shape.^1^ This ability has driven their use in biolabeling,^2,3^ artificial lighting,^4^ lasers,^5^ photovoltaics,^6^ and infrared detectors.^7^ However, the small size of the cQDs also leads to undesired fluctuations in their optical behavior. In particular, studies of individual cQDs have revealed several phenomena in their emission spectra. First, individual nanocrystals “blink”, meaning that they switch between periods of efficient and inefficient fluorescence.^8^ Second, the emission wavelength of single cQDs varies over time, referred to as spectral diffusion.^9,10^ Both of these phenomena are not directly visible in ensemble measurements of cQDs due to averaging. Nevertheless, blinking decreases the average emission efficiency, and spectral diffusion contributes to line width broadening. While optimization of cQD syntheses has reduced these effects, they have not yet been eliminated.^11−14^ Further improvements can be guided by a detailed understanding of the underlying photophysical processes in cQDs. Because blinking and spectral diffusion vary from particle to particle, experiments on individual cQDs are helpful for gaining insights.

Different mechanisms have been identified to cause blinking. A common explanation is the temporary charging of nanocrystals after photoexcitation, forming a weakly emissive trion.^8,13^ Also, trapping of hot^15^ and band-edge excitons^16^ has been shown to play a significant role. On the other hand, spectral diffusion is commonly attributed to fluctuating electric fields due to unbalanced charges in the vicinity of the nanocrystal, polarizing the exciton and shifting the emission wavelength.^17^ This understanding of the origin of blinking and spectral diffusion was gained by investigating the emission spectrum and the excited-state lifetime. However, such studies predominantly provide insights into the lowest excited level.

Measurements of dynamic fluctuations of higher excited levels promise complementary insights into the dynamics of the nanocrystals. Unfortunately, modest absorption cross sections render direct absorption measurements difficult for individual quantum emitters at room temperature. While techniques such as balanced detection of signal and reference beams^18−20^ or spatial modulation^21,22^ and careful minimization of background scattering can suppress technical noise sources, shot noise poses a fundamental limit to the minimally required integration time. It prevents the rapid measurement of single-particle extinction spectra at room temperature.

Photoluminescence excitation (PLE) spectroscopy overcomes this limitation by indirectly monitoring the absorption process in emissive samples through detection of the subsequent red-shifted photon emission. Because of reduced shot noise, the background-free detection scheme requires a significantly lower photon budget than direct methods (see the calculation in Section S1 of the Supporting Information). Hence, single-emitter PLE spectroscopy is commonly applied to organic molecules^23−25^ and epitaxially grown quantum dots^26^ at cryogenic temperatures. Furthermore, PLE has also been applied to study excited states in colloidal nanocrystals at cryogenic temperatures.^27−29^ However, because temporal fluctuations in the optical properties of individual emitters severely hamper the measurement of excitation spectra,^30^ which require sequential measurements to probe different wavelengths, it is still a niche technique in the cQD field, especially at room temperature. Other approaches include: (i) Fourier excitation spectroscopy^31−33^ and (ii) comparison of the optical response from a wavelength-tunable laser with one from a reference laser at a fixed wavelength.^34^ While these allow construction of a time-averaged excitation spectrum of the brightly emitting state, the typical measurement lasts from minutes^30−32,34^ down to half a second per scan,^33,35^ making them too slow to resolve intermittent effects fully. To date, no method provides excitation spectra fast enough to resolve the dynamic processes in individual emitters or to classify instances when the emitter is in a specific state of interest.

Here, we investigate processes responsible for blinking and spectral diffusion in CdSe cQDs by rapidly measuring a series of excitation spectra of individual quantum emitters. Simultaneously, we recorded emission spectra and fluorescence lifetime to track fluctuations of optical properties and identify bright, gray, and dim emission periods. We find significant differences in the excitation spectra collected during the bright and gray periods. A closer examination of the excitation-peak areas and spectral positions points toward a charging mechanism. Excitation spectra acquired during dim periods closely resemble spectra of bright periods, indicating that trapping, rather than charging, is responsible for these observations. We further show that, when the emission peak spectrally diffuses to lower energy during bright periods, the radiative decay rate decreases while the oscillator strength of higher excited states is increased. Effective-mass calculations show that a few elementary charges on the surface of cQDs can create sufficiently strong electric fields to significantly alter the oscillator strengths of several excited states.

We investigate colloidal core/shell/shell CdSe/CdS/ZnS quantum dots that emit around 2 eV (black line in Figure 1a). Section S2 provides further details about the samples and their preparation. The excitation spectrum of the ensemble of these cQDs consists of the lowest-energy peak, which is slightly blue-shifted from the emission, and additional peaks due to higher excited states (blue line in Figure 1a). These higher-energy peaks can be assigned to transitions between distinct electron and hole levels by comparing with previous calculations,^36^ as indicated in Figure 1b.

**Figure 1:**
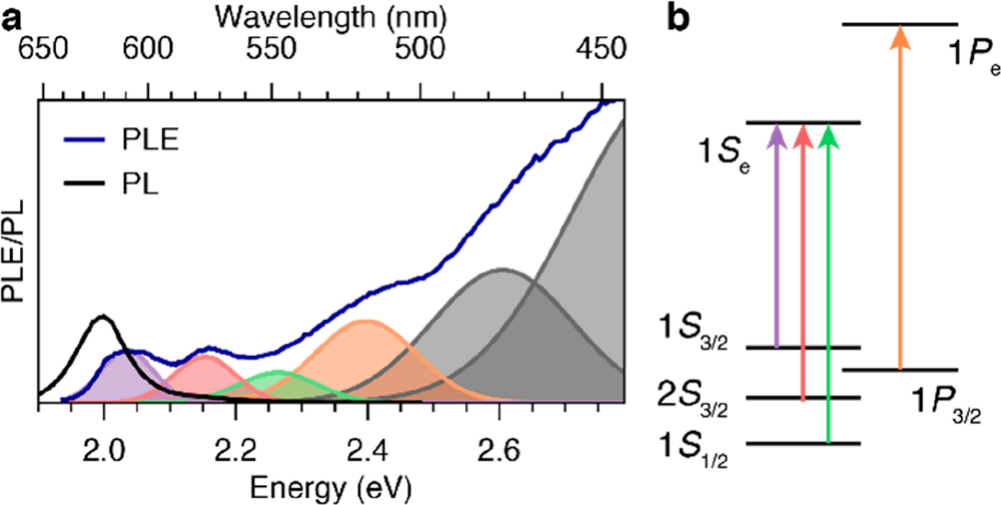
Room-temperature photoluminescence excitation (PLE) and emission (PL) spectra of an ensemble of CdSe/CdS/ZnS core/shell/shell cQDs. (a) The PL spectrum, excited with a wavelength of 405 nm, features a single emission peak. The PLE spectrum consists of a series of peaks at higher energies that can be disentangled by fitting with a series of Gaussian functions. (b) Well-resolvable near-band-edge peaks in the PLE spectrum can be assigned to distinct electron–hole transitions, indicated with different colored vertical arrows.

While an ensemble of the studied cQDs displays a constant emission intensity and photon energy, these properties fluctuate for individual cQDs. In the representative single-particle intensity trace in Figure 2a, periods of bright emission with around 450 detected counts per 10 ms are interrupted at random intervals by periods with significantly less than 100 counts per 10 ms. The stochastic nature of such blinking and spectral diffusion detrimentally affects the measurement of single-particle PLE spectra because, in contrast to photoluminescence (PL) spectra, all wavelengths have to be probed sequentially.

**Figure 2:**
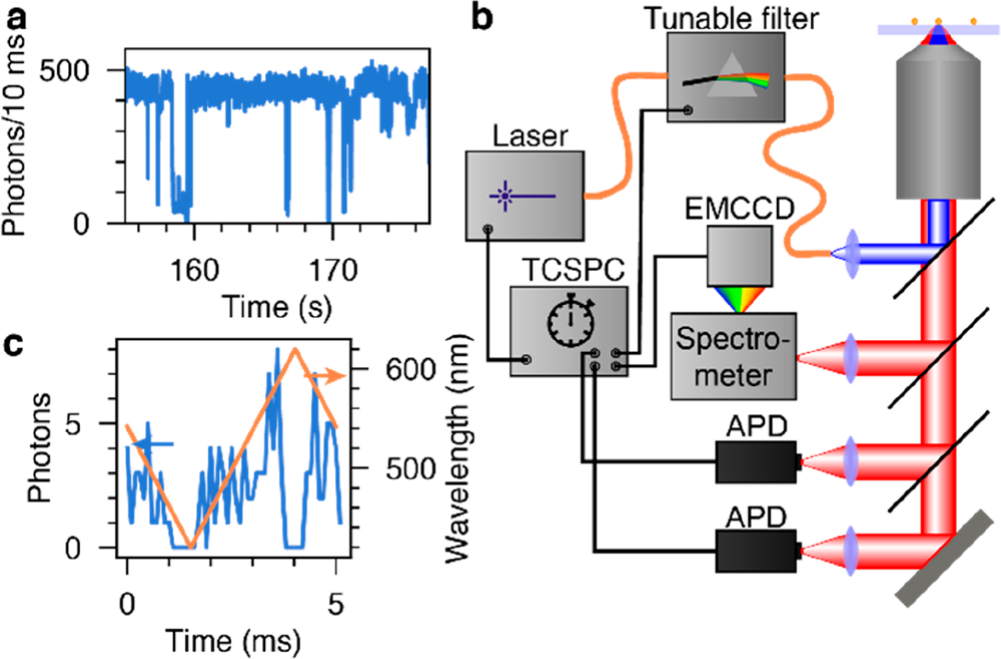
Room-temperature measurement of PLE spectra of blinking cQDs. (a) An individual cQD occasionally emits only weakly. These events can last for less than a millisecond or for tens of seconds. (b) To acquire high-quality PLE spectra that can circumvent these optical instabilities, we rapidly and repeatedly modulate the excitation wavelength of a broadband pulsed laser source using a tunable filter consisting of a galvo-mirror and a diffraction grating. The wavelength-modulated laser excites an individual cQD. Its emission is partially directed to a grating spectrometer in front of an electron-multiplying charge-coupled device (EMCCD) camera to record emission spectra. The other half is sent to two avalanche photodiodes (APDs) in a Hanbury-Brown–Twiss configuration. All events are tracked and synchronized using time-correlated single-photon counting (TCSPC) electronics. (c) For each detected photon, the excitation wavelength (orange line) is encoded in the arrival time relative to the start of the last galvo-mirror cycle.

To circumvent this problem, we rapidly and repeatedly sweep the excitation wavelength over the spectral range of interest while continuously recording the emission of a single cQD. This is achieved by spectrally filtering the broadband output of a pulsed supercontinuum laser with a galvo-mirror and a diffraction grating (schematically depicted in Figure 2b with details and calibrations in Sections S2 and S3). We selected the galvo-mirror scan rate such that one cycle (forward plus backward scan) lasted 5 ms, leading to a total of 200 forward and 200 backward scans per second (or 400 excitation scans in total per second). The filtered beam is coupled into a microscope to excite an individual cQD with a fluence generating less than one exciton per pulse (Section S4). This configuration allows for the measurement of hundreds of excitation spectra per second. While an individual spectrum represents a relatively small number of photons (Figure 2c), spectra obtained when the cQD is in the same state (i.e., with similar emission properties) can be averaged to increase the signal-to-noise ratio. To facilitate this, we collected two other pieces of information simultaneously with the PLE spectra. We recorded PL spectra with a grating spectrometer and PL lifetimes with time-correlated single-photon counting.

The chaotic intensity and lifetime traces of an individual cQD can be represented as a fluorescence lifetime intensity distribution (FLID) map, as shown in Figure 3a.^37^ The clustering of events at specific lifetimes and intensities reveals three distinct emission characteristics. The predominantly observed bright emission periods are accompanied by a PL lifetime of 27 ns. They are interrupted by gray periods, which give 50 counts/10 ms and a lifetime of 1.7 ns, and dim periods, which give 5 counts/10 ms and a lifetime of 0.6 ns. Observations outside the identified clusters can be caused either by switching of the quantum dot at time scales shorter than the binning time or by a continuously varying non-radiative decay rate.^16^

**Figure 3:**
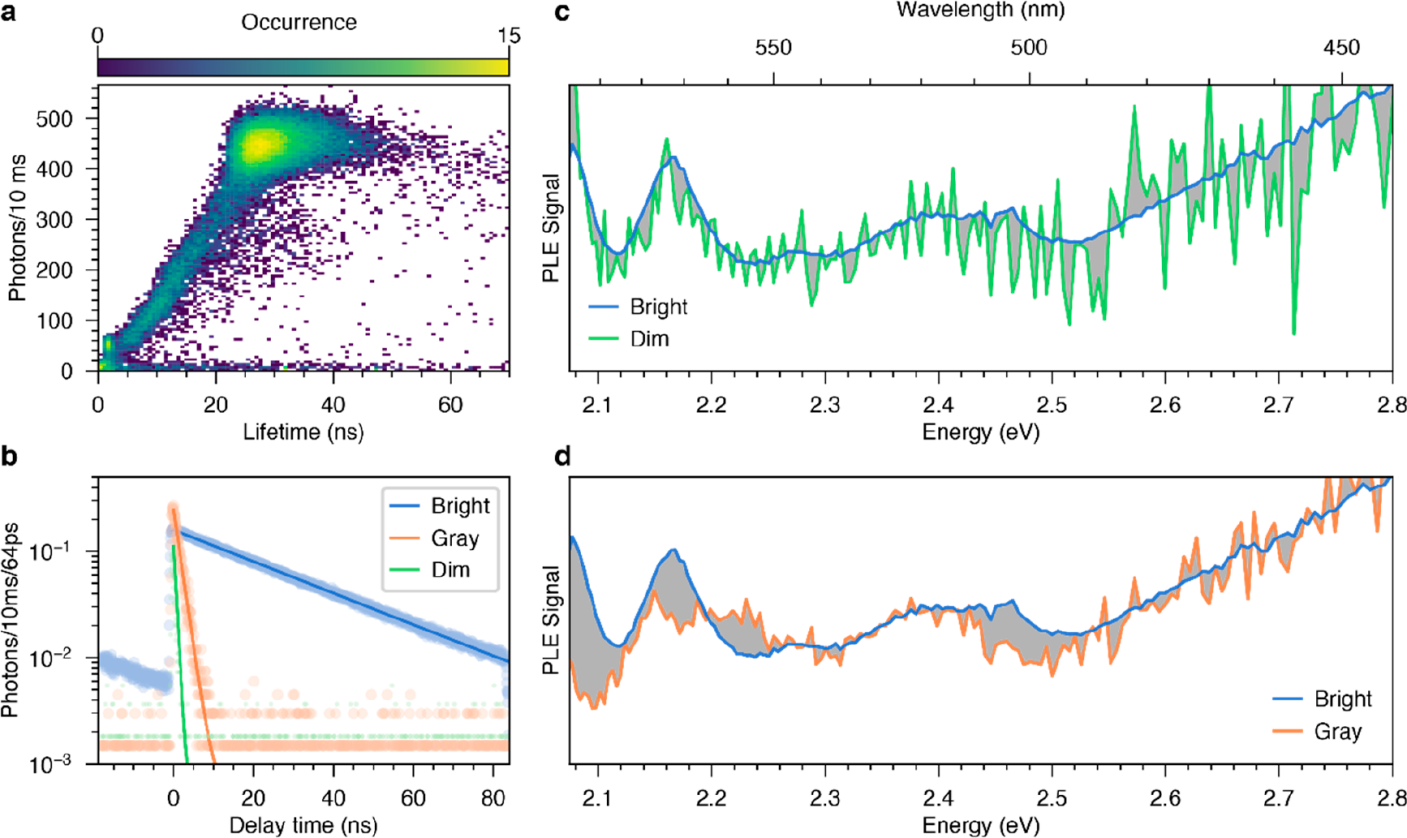
Room-temperature PLE spectroscopy of an individual, blinking cQD. (a) A fluorescence lifetime intensity distribution (FLID) map is obtained by correlating the lifetime and intensity of the binned photon stream. The cQD switches between bright, gray, and dim emission, as seen by clusters of occurrence. (b) Decay traces during bright, gray, and dim periods. For the gray periods, the increased PL intensity at zero delay time suggests that the cQD is charged. (c) The PLE spectra during bright and dim periods look similar (within our noise). (d) During gray periods, the PLE spectrum is significantly altered. Differences in the spectra in panels c and d are shaded in gray.

We then selectively evaluated the photon stream only for periods during which the cQD is bright, gray, or dim. Figure 3b displays PL decay traces obtained for the three emission characteristics. Each trace exhibits a single-exponential decay, indicating that the excitation wavelength (which rapidly changes during the measurement) has no effect on the decay dynamics. During gray periods, the cQD shows an increased initial amplitude of the decay curve (at a delay time of 0 ns), indicating a faster radiative decay rate.^11^ This is consistent with the presence of an excess charge carrier that provides an additional pathway for radiative recombination.^15^ For the dim periods, our fit estimates a lifetime of 600 ps, close to the instrument response of our lifetime setup. Therefore, the initial amplitude cannot be used to extract the radiative decay rate.

While our analysis of the initial amplitude in lifetime measurements can provide evidence of charging, it does not reveal the sign of the excess charge carrier. However, identifying which charge carrier causes gray periods is important for preventing this blinking mechanism. Charging can also potentially affect the oscillator strength of higher excited states. Accordingly, we investigated the effect of fluorescence blinking on the excitation spectra of nanocrystals. We used the same assignment of time bins into bright, gray, and dim periods to isolate their time-averaged excitation spectra. Hence, while each of our rapid excitation scans (400 per second) provides at most 100 photons (Figure 2c), selecting time bins and aggregating the excitation scans yields PLE spectra with sufficient signal-to-noise ratio. We observe that the PLE spectra above 2.6 eV differ only in amplitude but not in shape. This indicates that differences in the signal are caused only by a varying quantum yield (QY) and not by changes in the absorption. To obtain insight into modified absorption at lower photon energies, we thus used the high-energy signal to normalize out differences in QY in the different periods. In Figure 3c, we show excitation spectra of the single cQD constructed from the bright (blue) and dim (green) periods. The latter is noisy because dim periods are rare, and the corresponding photon count is low (only 7% of the collection time, resulting in 5000 photons). Apart from the noise and imperfect background correction, the spectra were very similar.

In contrast, comparing the PLE spectra obtained during the bright and gray periods reveals significant differences (Figure 3d). While the high-energy portion of the band-edge excitation peak (1*S*_e_1*S*_3/2_) is visible during bright periods below 2.1 eV, we only observe the onset of this peak during the gray periods. Gray periods also exhibit a significantly reduced peak around 2.17 eV compared to bright periods, accompanied by enhanced absorption around 2.23 eV. The peak at 2.4 eV appears to be shifted to lower energy and slightly reduced. The overall reduction in signal of the higher excited state suggests negative charging. Charging with an excess electron partially blocks absorption into all of the excitonic states involving the 1*S*_e_ electron level, as reported for ensembles of cQDs that were negatively charged by chemical^38−42^ or electrochemical^43−45^ methods.

So far, we have focused on the origin of periods of decreased emission intensities. We now turn our attention to spectral fluctuations that occur while the cQD remains bright. Figure 4a shows a time series of PL (below 2.07 eV) and PLE spectra (above 2.07 eV) of an individual cQD. The emission peak energy fluctuates with jumps of up to 45 meV. When the emission shifts to a lower energy, the excitation peak around 2.12 eV broadens. To quantitatively analyze this observation, we sorted the time bins by their peak emission energy. We only considered time bins during which the emission was bright. The sorted emission spectra are shown in Figure 4b. For lower emission energies, the emission peak becomes increasingly broad while retaining the same area, indicating that the overall quantum yield remains constant during spectral diffusion in bright periods (Section S6). Simultaneously, the fluorescence lifetime increases significantly as the emission shifts to lower energy, almost doubling for the reddest emission (Figure 4c). Because of the high quantum yield, the total decay rate well approximates the radiative decay rate (Section S6) and can be taken as a proxy for the band-edge oscillator strength (lower points in Figure 4e).

**Figure 4:**
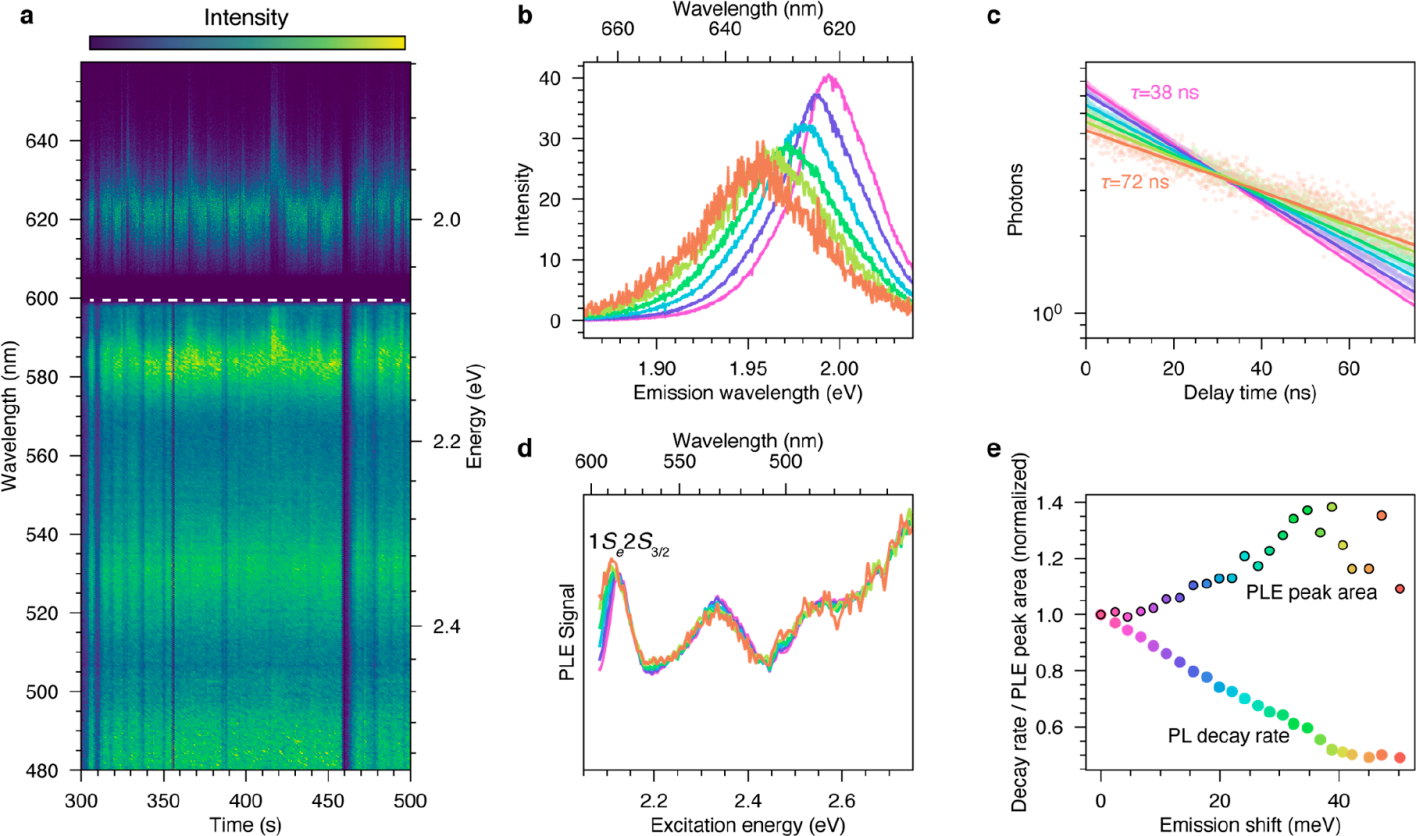
Room-temperature spectral diffusion of an individual cQD. (a) A time series of emission spectra (below 2.07 eV) and excitation spectra (above 2.07 eV, edge of short-pass filter indicated by white dashed line) of a single cQD. The spectra reveal fluctuations in the emission peak position and correlated changes in the PLE spectra. (b) The PL data are time-binned and then sorted according to the peak emission wavelength in the respective time bin. Lower-energy emission peaks are broadened. (c) Evaluation of the fluorescence decay of sorted data shows that the lifetime is prolonged when the emission is at lower energy. (d) PLE spectra after sorting by peak emission energy. The excitation peak at 2.12 eV increases in area as the emission shifts to lower energy. (e) Fluorescence decay rate and area under the excitation peak at 2.12 eV in the same cQD after sorting by peak emission energy. A strong correlation is seen. Both quantities are normalized to the value obtained for the highest emission energy. The data in panels b–e share the same color scheme.

We can also use the excitation spectra to investigate possible changes in the oscillator strength of higher excited states during spectral diffusion. The PLE spectra, grouped by emission peak energy, are shown in Figure 4d. The excitation spectra remain identical above 2.6 eV during spectral diffusion. However, an increase in the area of the 1*S*_e_2*S*_3/2_ peak at 2.12 eV highlights a growing oscillator strength in this spectral region as the emission shifts to lower energies (upper points in Figure 4e). The linear trends for the extracted oscillator strengths for the first and second excited states, which are decreasing and increasing, respectively, as indicated by Figure 4e, suggest that oscillator strength is transferred from the band-edge state to a transition near 2.12 eV. Indeed, extrapolating the linear trends reveals that the absorption feature near 2.12 eV would approximately double in area at the zero decay rate. From our analysis of ensemble spectra (Figure 1a), we know that the 1*S*_e_1*S*_3/2_ and 1*S*_e_2*S*_3/2_ peaks have comparable areas, and therefore, the total oscillator strength is conserved. Thus, what is the mechanism for transfer of oscillator strength during spectral diffusion?

To rationalize this transfer of oscillator strength, we modeled the effect of electric fields caused by excess surface charges on the oscillator strength of the different transitions. We used a simplified two-band effective-mass model^46,47^ and calculated the effects of a homogeneous electric field with a variational approach. Details of the model and an analysis of numerical convergence can be found in Sections S7 and S8. The drop in band-edge oscillator strength as the emission shifts to lower energy, as predicted by the effective-mass model, is plotted against the experimentally determined oscillator strength in Figure 5a. The model and experiment are in good agreement, both showing a drop to half of the initial value at a Stark shift of 40 meV. Such a change requires a homogeneous electric field of ∼10 MV/m, approximately the field created by two elementary charges on the nanocrystal surface (Section S9).

**Figure 5:**
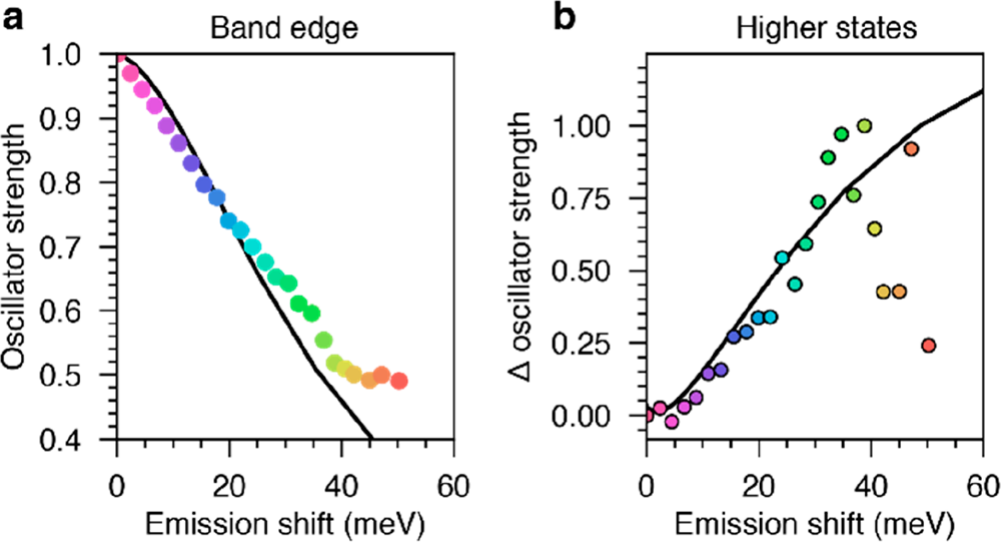
Comparison of the effective-mass model (lines) to experimental results (filled circles). (a) The loss of band-edge oscillator strength is plotted against the shift of the emission peak to lower energy. (b) The increase in the oscillator strength of transitions around 2.12 eV is plotted against the shift of the emission peak to lower energy. The increase in oscillator strength is normalized to the value obtained at the largest shift where data extraction was still reliable. The data points in panels a and b share the same color scheme as in Figure 4e.

The high-energy part of the 1*S*_e_2*S*_3/2_ peak shown in the PLE spectra in Figure 4d remains almost unchanged. This suggests that all spectral changes can be explained by a near-constant 1*S*_e_2*S*_3/2_ peak and an additional slightly red-shifted peak that grows in area as the emission shifts to lower energy. Indeed, our calculations show that the red shift of the 1*S*_e_2*S*_3/2_ state should be much smaller than that for the 1*S*_e_1*S*_3/2_ state, and its oscillator strength should vary only slightly. We suggest that, as the emission shifts to lower energy, the main change of the excitation spectrum is the increased presence of a 1*S*_e_1*P*_3/2_ absorption that spectrally overlaps with the 1*S*_e_2*S*_3/2_ feature. However, appropriate treatment of the oscillator strength of the higher excited states is more challenging because our calculations severely underestimate the relative oscillator strength of the 1*S*_e_2*S*_3/2_ transition in comparison to experimental data. Hence, our modeled data cannot be meaningfully normalized to the zero-field value, and the oscillator strengths cannot be directly compared to the experimental data. To compare the simulation to our experimental data, we thus plot the change in oscillator strength integrated over the 1*S*_e_1*P*_3/2_ and 1*S*_e_2*S*_3/2_ states and normalize this by the change in oscillator strength at the largest Stark shift of around 40 meV. This is the largest shift where fitting of the experimental PLE spectrum yields satisfactory results. Figure 5b illustrates that our effective-mass model matches the increase in the oscillator strength of the second excited-state manifold with the shift of the emission. This suggests that the experimentally observed broadening and increase in oscillator strength are caused by the 1*S*_e_1*P*_3/2_ transition that is parity forbidden but becomes increasingly allowed in an external electric field and leads to pronounced induced absorption.

The quantum-confined Stark effect, which shifts the emission to a lower energy and reduces the radiative decay rate, is commonly accepted as the reason for spectral diffusion. However, while induced absorption caused by uniform electric fields has been predicted,^48,49^ it has only been observed as a relatively weak effect in measurements using externally applied fields and on ensembles of nanocrystals.^50,51^ Our experimental observation of induced absorption during the random spectral diffusion of an individual nanocrystal suggests that single elementary charges on the nanocrystal surface can generate sufficient electric fields to severely alter absorption spectra beyond simple shifts of the energetic position of the transitions. All experimentally observed fluctuations during bright emission periods (emission shift to lower energy, prolonged lifetime, and induced absorption) can be explained by a fluctuating, uniform electric field with a field strength of around 10 MV/m inside the cQD. Indeed, electric fields of this magnitude can be caused by individual elementary charges located at the surface of the cQD (Section S9). The correlation between the emission shift, drop in the radiative decay rate, and increased oscillator strength of higher transitions is further proof that spectral diffusion in nanocrystals is caused by fluctuating electric fields.

Fluctuations in the optical properties of quantum emitters represent an intriguing phenomenon that has been extensively studied by recording the time-resolved emission intensity, spectra, and fluorescence decays. While insight into the fluctuation of optical properties of higher excited states can further the understanding and aid in model validation, technical limitations have largely prohibited such measurements. We demonstrated that rapid scanning of the excitation wavelength can track fluctuations in the excitation spectrum of CdSe quantum dots and thereby provide information about the effects of blinking and spectral diffusion. Our excitation spectroscopy revealed two types of blinking in the same quantum dot. Only one type was accompanied by a modified excitation spectrum and can therefore be attributed to charging. The investigated nanocrystals also showed spectral diffusion during the bright periods. Time-resolved excitation spectroscopy revealed that a red shift of the emission is accompanied by a transfer of oscillator strength from the emitting state to higher excited states. Our experimental data indicate a strong impact of individual charge carriers on the photoluminescence excitation spectra of the individual nanocrystals. The observed effects have implications on applications involving energy transfer, e.g., in bioimaging, but they could also aid in future improvements of light-emitting devices. Our rapid acquisition of a large number of excitation spectra further brings the under-represented excitation spectroscopy closer to the versatility of emission spectroscopy in both spectral and temporal resolution.

## Supporting Information Available

Description of the shot-noise analysis (Section S1), materials and methods (Section S2), calibration procedure for experimental apparatus (Section S3), estimation of exciton occupancy (Section S4), quantum yield of studied cQDs (Section S5), evidence for constant quantum yield during spectral diffusion (Section S6), calculation of excited states in an electric field (Section S7), convergence of calculations (Section S8), and estimate of the electric field generated by an elementary charge (Section S9) (PDF)
