# Supplementary material for: Fluctuations in the Photoluminescence Excitation Spectra of Individual Semiconductor Nanocrystals
Source: J Phys Chem Lett. 2024 Apr 29;15(18):4844–50. doi: 10.1021/acs.jpclett.4c00516 (PMC11089566; doi:10.1021/acs.jpclett.4c00516)
Supplement: Supplementary file 1 — jz4c00516_si_001.pdf [file jz4c00516_si_001.pdf]

Supporting Information for

Fluctuations in the Photoluminescence Excitation Spectra of Individual Semiconductor Nanocrystals

Robert C. Keitel,[†] *Raphael Brechbühler,*[†] *Ario Cocina,*[†] *Felipe V. Antolinez,*[†] *Stefan A. Meyer,*[†]

Sander J. W. Vonk,[†,§] *Henar Rojo,*[†] *Freddy T. Rabouw,*[†,§] *and David J. Norris**[,†]

[†]Optical Materials Engineering Laboratory, Department of Mechanical and Process Engineering, ETH Zurich, 8092 Zurich, Switzerland

[§]Debye Institute for Nanomaterials Science, Utrecht University, 3584 CC Utrecht, The Netherlands

Contents

Section S1. Shot Noise in Excitation and Absorption Spectroscopy S2
Section S2. Materials and Methods S2
Section S3. Calibration of the Excitation Spectroscopy Setup S4
Section S4. Estimation of Exciton Occupancy S6
Section S5. Quantum Yield of the Studied cQDs S7
Section S6. Proof of Constant Quantum Yield During Spectral Diffusion S8
Section S7. Calculation of Excited States of cQDs in an Electric Field S9
Section S8. Convergence of the Calculations S12
Section S9. Field Generated by an Elementary Charge S13
References S14

Section S1. Shot Noise in Excitation and Absorption Spectroscopy

The detected signal in absorption spectroscopy is given by the number of photons absorbed: $N_{\mathrm{abs}} = A_{\mathrm{abs}} N_{\mathrm{inc}}$, where N_{inc} is the number of incident photons, A_{abs} gives the fraction of absorbed photons, and N_{abs} is the number of absorbed photons. However, the shot noise is determined by the square root of the number of photons arriving at the detector, $\sqrt{N_{\mathrm{inc}} - N_{\mathrm{abs}}}$, which for a small fraction of absorbed photons is well approximated by $\sqrt{N_{\mathrm{inc}}}$. Accordingly, for small absorption cross sections, even when eliminating all technical noise sources, many absorbed photons are required to reach satisfactory signal-to-noise ratios. For example, if $A_{\mathrm{abs}} = 10^{-6}$, this means that 10^6 absorbed photons are required just to reach a signal-to-noise ratio of unity.

This fundamental limit can be overcome by indirectly probing the absorption process. While the heat generated following absorption of a photon can be used as a reporter,[S1,S2] this technique is mainly suited to non-emissive samples and requires suitable photothermal media.[S3] For emissive samples, photoluminescence excitation spectroscopy (PLE) is the method of choice. Here, rather than the absorption of a photon, the subsequent red-shifted emission is detected. The fraction of absorbed photons that lead to a detected emitted photon is given by the fluorescence quantum yield (QY) of the emitter and the optical detection efficiency. Even for pessimistic values of $\frac{N_{\mathrm{detected}}}{N_{\mathrm{absorbed}}} = 1\%$ only 10^2 photons have to be absorbed to reach a signal-to-noise ratio of unity, compared to the 10^6 needed for an absorption measurement. Accordingly, for emitters with a rather small absorption cross section and high quantum yield, excitation spectroscopy enables orders of magnitude faster measurements.

Section S2. Materials and Methods

Nanocrystal synthesis and sample preparation. CdSe cores with an average radius of 1.9 nm and a lowest-energy absorption peak at 585 nm were prepared by modifying the protocol published by Reiss *et al.*[S4] After purification of the cores, CdS and ZnS shells were grown in a separate step,[S5] during which a high-temperature annealing was used to smoothen the confining potential and release strain. A more detailed description was published by le Feber *et al.*[S6] After shell growth, the reaction mixture was allowed to cool to room temperature. The cQDs were twice purified by precipitation with methylacetate and dispersed in hexane.

Samples for room-temperature single-particle spectroscopy were prepared by diluting the stock dispersion by six orders of magnitude in hexane. 100 μl of the dilute dispersion were spin-coated onto a glass cover slip at 1000 rpm.

Single-particle optical spectroscopy. Our photoluminescence excitation spectroscopy setup is based on a pulsed supercontinuum fiber laser (NKT SuperK Fianium, repetition rate 10 MHz). The collimated output of the laser was reflected off two mirrors (Thorlabs, BB1-E02) and aligned into an expanding telescope, consisting of a pair of lenses (Thorlabs, AC127-019-A-ML and AC254-150-A-ML). We intentionally used dielectric mirrors with a reduced transmission in the infrared (IR) spectral range to suppress the unwanted IR output of the fiber laser. Another pair of mirrors directed the beam onto a galvo mirror (Thorlabs, GVS011/M). From this, the beam was passed through a transmissive diffraction grating (Thorlabs, GT25-06V) and coupled by a lens (Thorlabs, AC254-030-A-ML) into a single-mode fiber (Thorlabs, P3-460B-FC-5). The grating, in combination with the lens, coupled only a narrow band of the spectrum into the fiber. The galvo-mirror angle controled the center wavelength of the transmitted band. To maintain high coupling efficiency over a broad range of wavelengths, it is important to keep the distance between galvo mirror and grating as small as possible. The galvo-mirror angle was controlled via an applied voltage. For our experiments, we generated a triangle waveform (Agilent 33521A) at 200 Hz with suitable amplitude and offset to cover the entire spectral range of interest. Because of the finite bandwidth of the galvo mirror, a triangle waveform was rounded-off at the extreme points, which corresponds to the end of the scan range. For this reason, we chose the scan range slightly larger such that the range of interest was entirely in the linear part of the galvo mirror scan. The calibration steps necessary to obtain excitation spectra from the tunable filter can be found in Section S3. The optical output of this setup consisted of wavelength-modulated laser pulses, while the electrical output was a series of transistor–transitor logic (TTL) pulses that marked the beginning of each wavelength sweep. This electrical output was connected to one of the marker channels of a time-tagger box (Picoquant, Hydraharp 400).

The optical output was collimated, passed through a shortpass filter (Thorlabs, FESH0600), attenuated by absorptive neutral density filters (Thorlabs, NE10A-A), and coupled into an optical microscope (Nikon, Ti-U Eclipse). The excitation light was reflected off a 10/90 beam splitter (Thorlabs, BSN10R) and focused by an oil immersion objective (Nikon, Plan APO 100x VC, NA 1.4) onto the sample. Fluoresence was collected through

the same objective and passed through the beamsplitter. A tunable longpass filter was used to remove reflected excitation light (SEMROCK, VersaChrome TLP01-628). Half of the emission was reflected off a non-polarizing 50/50 beam splitter, focused (with a focal length of 200 mm) onto the entrance slit of an imaging spectrometer (Andor, Shamrock 303i), and imaged with an electron-multiplying charge-coupled device (EMCCD) camera (Andor, iXon 888 Ultra). The camera gave out a TTL pulse at the beginning of each frame that was also connected to the time-tagger box to correlate excitation and emission spectra. The other half of the emission was sent to a Hanbury-Brown-Twiss setup consisting of a non-polarizing 50/50 beam splitter and two nominally identical avalanche photodiodes (APDs, Excelitas SPCM-AQRH-14-TR). The output channels of the APDs were connected to the signal channels of the time-tagger box. Together with the laser sync signal connected to the sync channel of the time-tagger box, this allowed to record both the absolute photon arrival time and the delay after the excitation laser pulse, which was used to construct fluorescence decay curves.

Ensemble optical spectroscopy. PLE spectra of ensembles of cQDs were recorded from dilute dispersions in hexane using a fluorometer (Edinburgh Instruments FLS 980). Absorption spectra were collected with a spectrophotometer (Varian Cary 50). Samples were diluted in hexane and measured in a 1-cm path-length quartz cuvette. The ensemble quantum yield was measured using a Hamamatsu C11347 Quantaurus-QY spectrometer with an integrating sphere. Diluted samples were measured in a 1-cm path-length quartz cuvette.

Section S3. Calibration of the Excitation Spectroscopy Setup

For each detected photon, the time since the beginning of the last wavelength modulation cycle has to be mapped to the excitation wavelength. This mapping is based on two calibration steps. The arrival time of a photon first has to be converted to the voltage simultaneously applied to the galvo mirror. In principle, this is given by the waveform chosen to drive the galvo mirror. However, we measured the true waveform of the galvo mirror with an oscilloscope. Figure S1a shows that the deviation from the input can be described by two effects: (i) a rounding of the peak at the maximum of the curve and (ii) a constant time lag. During the slope, the actual waveform deviates from the input only by this lag. We found the lag, which is dependent on the frequency and amplitude of the waveform, by splitting the excitation spectra acquired on a single cQD in forward and backward scans and mapping them from the time to the voltage axis by the input function shifted by a lag. For a zero lag, the forward and backward scans are shifted with respect to each other, as can be seen in the dashed lines in Figure S1b. We

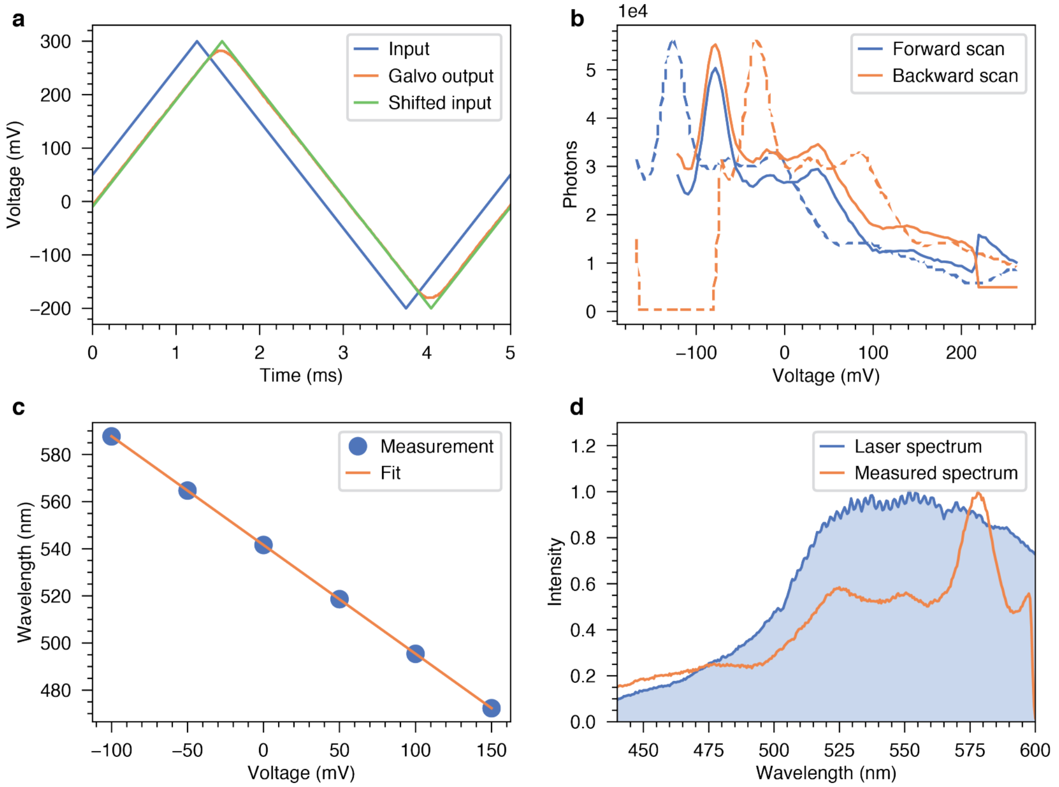

Figure S1. Calibration of excitation spectroscopy setup. (a) Deviation of the galvo-mirror output from the input due to finite bandwidth. (b) Obtained excitation spectra before (dashed lines) and after correction (solid lines). The corrected backward scan is vertically displaced for clarity. (c) Measured center wavelengths for different galvo-mirror voltages reveal a linear relationship. (d) Typical measured photoluminescence excitation spectrum from a single cQD before correcting for the wavelength dependence of the excitation laser (shown as laser spectrum).

reassign the times to voltages for different time shifts, and for each, calculate the product of the forward and the backward scan. The only difference to the formal definition of the cross-correlation is that we apply the timeshift symmetrically rather than to just one of the two scans. The product of the shifted spectra is our figure-of-merit for overlap of the forward and backward scans. The optimal time shift is found when the product is maximized. This calibration step was carried out once at the beginning of each experiment.

The resulting spectra are shown in Figure S1b in solid lines. The conversion from galvo-mirror voltages to center wavelengths of the transmitted light is dependent on the exact alignment of the filter box. We set the galvo-mirror input to different voltages and recorded the center wavelength with the same spectrometer used for the spectral measurements of the quantum dots. The relationship between voltage and wavelength is well-described by a linear function in the range of interest as seen in Figure S1c. With the obtained conversion function the data shown in Figure S1b can be plotted on a wavelength axis (Figure S1d).

The last step to obtaining a proper excitation spectrum is to record the spectrum of the excitation laser used. For this, we drove the galvo mirror with the triangle waveform and sent the fiber output onto our spectrometer. If the integration time is sufficiently long, this yields the spectrum of the excitation laser, shown as the blue shaded area in Figure S1d. The raw excitation spectrum had to be corrected for a flat background due to dark counts and divided by the measured reference spectrum. We stress that the output spectrum of the SuperK Fianium depends on the power and repetition rate chosen. Also, neutral density filters can have an undesired wavelength dependence in their transmission. Therefore, it is crucial to collect the reference spectrum under the same conditions as the experimental data.

Section S4. Estimation of Exciton Occupancy

To analyze the PLE spectra quantitatively, it is critical that the number of photons emitted is proportional to the number of photons absorbed. In typical quantum dots, while the exciton has a high quantum yield, the emission from the biexciton is much less efficient due to fast Auger recombination. As a result, the detected photon count is only proportional to the excitation power, as long as the average number of excitons generated per pulse is significantly below unity. To estimate the exciton occupancy for experimentally used excitation parameters, we first estimated the relative biexciton quantum efficiency (QY_{BX}/QY_X), where QY_{BX} and QY_X are the biexciton and exciton quantum yields, respectively. Figure S2a shows the second-order photon correlation of a single particle from the batch investigated. The fit reveals that $QY_{BX}/QY_X = 0.07$. We fixed the excitation wavelength to 495 nm, placed an individual cQD in focus, and varied the excitation power using neutral density filters with optical densities (OD) in the range of 0 to 3. For each excitation intensity, we estimated the photon emission rate by binning into 10 ms bins and histogramming. The photon count rate is then given as the position of the high-

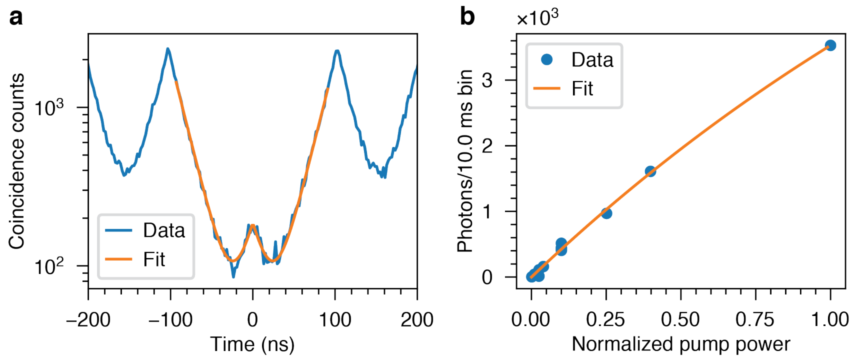

Figure S2. Estimation of exciton occupancy. (a) Second-order photon correlation revealing $QY_{BX}/QY_{BX} = 0.07$. (b) Saturation curve and fit to a model, taking into account the estimated QY_{BX}.

intensity peak in the histogram. This analysis removes the power-dependent blinking dynamics. The obtained data shown in Figure S2b can be fitted with the expression:

$$I = a\left\{1 - P(0, b\,p) + \frac{\mathrm{QY_{BX}}}{\mathrm{QY_X}}\left[1 - P(0, b\,p) - P(1, b\,p)\right]\right\}, \tag{S1}$$

where p is the excitation power relative to the highest power used, I is the photon count rate, $P(i, j)$ the probability given by the Poisson distribution to find a value of i for a mean value of j, and a and b are fitting parameters. We estimate $b = 0.46 \pm 0.08$, which means that at maximum excitation power, we have an average exciton occupancy of 0.46. All experiments were conducted with an OD = 1 filter, so the average occupancy at 495 nm was around 0.046. For the analyzed cQDs and the spectrum of the laser, the highest count rate is up to four times of the count rate obtained at 495 nm. Therefore, the maximum occupancy is around 0.18, which is relatively high but still sufficiently in the linear regime to facilitate data interpretation. The parameter a gives the detected count rate due to single-exciton emission at saturation. This is estimated at 9460 ± 1430 counts per 10 ms, which, together with the experimental repetition rate of 10 MHz, yields a detection efficiency of slightly below 10%.

Section S5. Quantum Yield of the Studied cQDs

Absorption and excitation spectra can only be compared straightforwardly if the fluorescence quantum yield is independent of the excitation wavelength. To verify this, we measured the fluorescence quantum yield of a diluted dispersion of quantum dots from the same synthesis. For the particles studied, the fluorescence quantum yield shows no significant dependence on the excitation wavelength, as shown in Figure S3. Thus, the PLE signal should be proportional to the absorption at the respective energy.

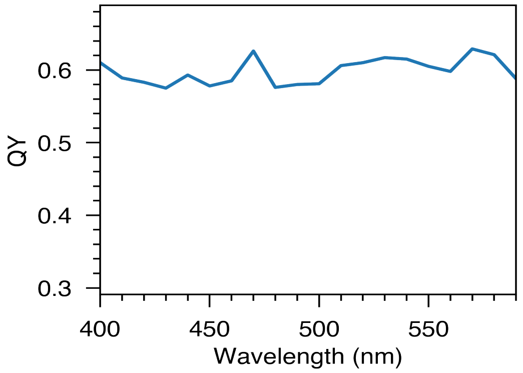

Figure S3. Wavelength-resolved quantum yield of the ensemble of CdSe/CdS/ZnS core/shell/shell cQDs studied.

Section S6. Proof of Constant Quantum Yield During Spectral Diffusion

We use the fluorescence decay rate as a proxy for the oscillator strength of the emitting transition in our analysis of spectral diffusion. This is only valid if the fluorescence decay rate is equal to the radiative decay rate. In other words, we must assume that $\Gamma_{\mathrm{tot}} = \Gamma_{\mathrm{r}} + \Gamma_{\mathrm{nr}} \approx \Gamma_{\mathrm{r}}$, where Γ_{tot}, Γ_{r}, and Γ_{nr} are the total, radiative, and nonradiative decay rates, respectively. This requires that $\mathrm{QY} = \frac{\Gamma_{\mathrm{r}}}{\Gamma_{\mathrm{r}}+\Gamma_{\mathrm{nr}}} \approx 1$ during periods of bright emission. Figure S4a shows that while the emission broadens from 23 to 36 meV during spectral diffusion, the integrated intensity stays almost constant and only fluctuates by less than 10%. However, because the emission spectra average over all excitation wavelengths and we observed increased oscillator strength of the absorbing transitions when the emission is red-shifted (see results in the main text), a drop in QY could be offset by the increase in oscillator strength.

To avoid this potential artifact, we analyzed the fluorescence decay when the sample was excited with wavelengths shorter than 510 nm for the periods of different emission peak wavelengths. Figure S4b shows the decay rates and amplitudes (which are proportional to the radiative decay rate)[S7] obtained for the different emission peak positions. Note that decay amplitudes were corrected by a factor $1 - e^{-\frac{T}{\tau}}$, where T is the duration between excitation pulses and τ the estimated lifetime, to correct for pile-up due to the high repetition rate of the laser. The data points can be well fitted by a linear function, which indicates that the non-radiative decay time τ_{nr} does not

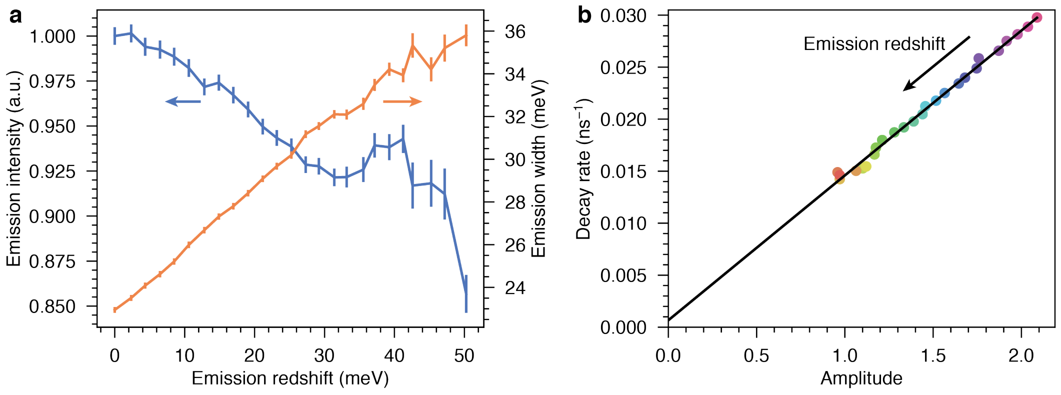

Figure S4. Constant QY during spectral diffusion. (a) As the emission spectrum broadens, the integrated emission intensity fluctuates by only 10% as the peak position shifts. (b) A linear fit (solid line) of the experimental relationship between decay rate and amplitude for different emission peak positions (color-coded dots) indicates that the QY is near unity. Only photons emitted when the sample was excited with wavelengths shorter than 510 nm have been considered to suppress a correlating effect of spectral diffusion.

change during spectral diffusion. The intercept of the fit indicates a non-radiative decay rate of 6.7×10^{-4} ns^{-1} and a QY above 95% for all emission-peak positions.

Section S7. Calculation of Excited States of cQDs in an Electric Field

The effects of an external electric field on energy levels and oscillator strengths were calculated using an effective-mass model similar to that in the literature.[S8,S9] We considered the effects of confinement, the Coulomb interaction between charge carriers, and a uniform electric field. The Hamiltonian for the exciton is:

$$H = H_0^{\mathrm{e}}(\boldsymbol{r}_{\mathrm{e}}) + H_0^{\mathrm{h}}(\boldsymbol{r}_{\mathrm{h}}) + V_{\mathrm{C}}(\boldsymbol{r}_{\mathrm{e}}, \boldsymbol{r}_{\mathrm{h}}) - eV_{\mathrm{ext}}(\boldsymbol{r}_{\mathrm{e}}) + eV_{\mathrm{ext}}(\boldsymbol{r}_{\mathrm{h}}) , \quad \text{(S2)}$$

where $H_0^{\mathrm{e,h}}(\boldsymbol{r}_{\mathrm{e,h}})$ is the Hamiltonian for an electron or hole, respectively, in a spherical box, $\boldsymbol{r} = (r, \theta, \phi)$ is the spatial position in spherical coordinates, V_{C} is the potential due to the electron–hole Coulomb interaction, and V_{ext} is the potential due to a homogeneous electric field E_z in the *z* direction. We found solutions to eq S2 by a variational approach. As a basis, we used product wavefunctions formed by the single-particle wavefunctions:

$$\Psi^{(0)}_{n_{\mathrm{e}},l_{\mathrm{e}},m_{\mathrm{e}},n_{\mathrm{h}},l_{\mathrm{h}},m_{\mathrm{h}}}(\boldsymbol{r}_{\mathrm{e}}, \boldsymbol{r}_{\mathrm{h}}) = \psi^{\mathrm{e}}_{n_{\mathrm{e}},l_{\mathrm{e}},m_{\mathrm{e}}}(\boldsymbol{r}_{\mathrm{e}}) \psi^{\mathrm{h}}_{n_{\mathrm{h}},l_{\mathrm{h}},m_{\mathrm{h}}}(\boldsymbol{r}_{\mathrm{h}}) , \quad \text{(S3)}$$

which are the eigenfunctions of $H_0^{\mathrm{e}}(\boldsymbol{r}_{\mathrm{e}}) + H_0^{\mathrm{h}}(\boldsymbol{r}_{\mathrm{h}})$ with quantum numbers $n_{\mathrm{e}}, l_{\mathrm{e}}, m_{\mathrm{e}}$ and $n_{\mathrm{h}}, l_{\mathrm{h}}, m_{\mathrm{h}}$ for the electron and hole, respectively.

We assumed that holes are localized in the core and experience an infinitely high confining potential at the core radius, r_{CdSe}. We simplified the small conduction-band offset between CdSe and CdS by assuming that electrons are also delocalized through the CdS shell and experience the infinite potential at the CdS to ZnS interface at r_{CdS}. The electron single-particle wave functions are the solutions to the Schrödinger equation for H_0^{e}:

$$\psi^{\mathrm{e}}_{n_{\mathrm{e}},l_{\mathrm{e}},m_{\mathrm{e}}}(\boldsymbol{r}_{\mathrm{e}}) = \begin{cases} R_{n_{\mathrm{e}},l_{\mathrm{e}}}(r_{\mathrm{e}}) Y_{l_{\mathrm{e}}}^{m_{\mathrm{e}}}(\theta, \phi) & |r_{\mathrm{e}}| \le r_{\mathrm{CdS}} \\ 0 & \text{otherwise} \end{cases} . \quad \text{(S4)}$$

$R_{n_{\mathrm{e}},l_{\mathrm{e}}} = A_{n_{\mathrm{e}},l_{\mathrm{e}}} j_{l_{\mathrm{e}}}\left(\frac{\chi_{n_{\mathrm{e}},l_{\mathrm{e}}} r_{\mathrm{e}}}{r_{\mathrm{CdS}}}\right)$ is the radial wavefunction, and $Y_{m_{\mathrm{e}}}^{l_{\mathrm{e}}}(\theta, \phi)$ are the spherical harmonics. $j_{l_{\mathrm{e}}}(r)$ is the spherical Bessel function of order l_{e}, $\chi_{n_{\mathrm{e}},l_{\mathrm{e}}}$ its $n_{\mathrm{e}}^{\mathrm{th}}$ zero, and $A_{n_{\mathrm{e}},l_{\mathrm{e}}}$ is a normalization constant. The hole wavefunctions are obtained from eq S4 by replacing r_{CdS} with r_{CdSe}, and the subscript e by h. The solutions to the Schrödinger equation with eq S2 as the Hamiltonian are then written as linear combinations of the product functions defined in eq S3:

$$\Psi(\boldsymbol{r}_{\mathrm{e}}, \boldsymbol{r}_{\mathrm{h}}) = \sum_{n_{\mathrm{e}},l_{\mathrm{e}},m_{\mathrm{e}},n_{\mathrm{h}},l_{\mathrm{h}},m_{\mathrm{h}}} c_{n_{\mathrm{e}},l_{\mathrm{e}},m_{\mathrm{e}},n_{\mathrm{h}},l_{\mathrm{h}},m_{\mathrm{h}}} \Psi^{(0)}_{n_{\mathrm{e}},l_{\mathrm{e}},m_{\mathrm{e}},n_{\mathrm{h}},l_{\mathrm{h}},m_{\mathrm{h}}}(\boldsymbol{r}_{\mathrm{e}}, \boldsymbol{r}_{\mathrm{h}}) . \quad \text{(S5)}$$

To keep our notation compact, we label the product wavefunctions in the sum as $\Psi_{\mathrm{eh}}^{(0)}$, which just denotes the product formed from an electron and a hole state. The coefficients for the different eigenstates of the system are found by minimizing the energy expectation value

$$\langle E \rangle = \langle \Psi(\boldsymbol{r}_{\mathrm{e}}, \boldsymbol{r}_{\mathrm{h}}) | H | \Psi(\boldsymbol{r}_{\mathrm{e}}, \boldsymbol{r}_{\mathrm{h}}) \rangle\ , \tag{S6}$$

which constitutes solving the eigenvalues and eigenvectors of matrix **H** with elements

$$H_{ij} = \left\langle \Psi_{\mathrm{eh},i}^{(0)} \middle| H \middle| \Psi_{\mathrm{eh},j}^{(0)} \right\rangle. \tag{S7}$$

Due to the orthogonality of the single-particle wavefunctions, the operators $H_0^{\mathrm{e}}(\boldsymbol{r}_{\mathrm{e}})$ and $H_0^{\mathrm{h}}(\boldsymbol{r}_{\mathrm{h}})$ only appear on the matrix diagonal, and the values are given by the sum of the confinement energy:

$$H_{ii} = \frac{\hbar^2 \chi_{n_{\mathrm{e},i}, l_{\mathrm{e},i}}^2}{2 M_{\mathrm{e}} r_{\mathrm{CdS}}^2} + \frac{\hbar^2 \chi_{n_{\mathrm{h},i}, l_{\mathrm{h},i}}^2}{2 M_{\mathrm{h}} r_{\mathrm{CdSe}}^2}, \tag{S8}$$

with $\hbar$ the reduced Planck's constant and $M_{\mathrm{e,h}}$ the reduced masses of electron and hole.

The Coulomb interaction leads to off-diagonal terms that couple product wavefunctions with different quantum numbers. Neglecting effects due to the contrast in dielectric constant ε between the cQD and its environment or between core and shell, the potential due to the Coulomb interaction is given by:

$$V_{\mathrm{C}}(\boldsymbol{r}_{\mathrm{e}}, \boldsymbol{r}_{\mathrm{h}}) = \frac{-e^2}{4\pi\varepsilon\varepsilon_0 |\boldsymbol{r}_{\mathrm{e}} - \boldsymbol{r}_{\mathrm{h}}|}, \tag{S9}$$

where ε_0 is the vacuum permittivity. The position-dependent term can be expanded as a multipole:

$$\frac{1}{|\boldsymbol{r}_{\mathrm{e}} - \boldsymbol{r}_{\mathrm{h}}|} = \sum_{q=0}^{\infty} \sum_{p=-q}^{q} \frac{4\pi}{2q+1} \frac{\min(r_{\mathrm{e}}, r_{\mathrm{h}})^q}{\max(r_{\mathrm{e}}, r_{\mathrm{h}})^{q+1}} Y_q^p(\theta_{\mathrm{e}}, \phi_{\mathrm{e}}) Y_q^{p^*}(\theta_{\mathrm{h}}, \phi_{\mathrm{h}}) \tag{S10}$$

where the asterisk denotes the complex conjugate. The matrix entries of the coulomb interaction are then evaluated by:

$$\left\langle \Psi_{\mathrm{eh1}}^{(0)} \middle| \frac{1}{|\boldsymbol{r}_{\mathrm{e}} - \boldsymbol{r}_{\mathrm{h}}|} \middle| \Psi_{\mathrm{eh2}}^{(0)} \right\rangle =$$

$$\sum_{q=0}^{\infty} \sum_{p=-q}^{q} \frac{4\pi}{2q+1} \int_{\phi_{\mathrm{h}}=0}^{2\pi} \int_{\phi_{\mathrm{e}}=0}^{2\pi} \int_{\theta_{\mathrm{h}}=0}^{\pi} \int_{\theta_{\mathrm{e}}=0}^{\pi} \int_{r_h=0}^{r_{\mathrm{CdSe}}} \int_{r_e=0}^{r_{\mathrm{CdS}}} \Psi_{\mathrm{eh1}}^{(0)} \frac{\min(r_{\mathrm{e}}, r_{\mathrm{h}})^q}{\max(r_{\mathrm{e}}, r_{\mathrm{h}})^{q+1}} Y_q^p(\theta_{\mathrm{e}}, \phi_{\mathrm{e}}) Y_q^{p^*}(\theta_{\mathrm{h}}, \phi_{\mathrm{h}}) \Psi_{\mathrm{eh2}}^{(0)} r_{\mathrm{e}}^2 r_{\mathrm{h}}^2 \sin(\theta_{\mathrm{e}}) \sin(\theta_{\mathrm{h}})\, dr_{\mathrm{e}} dr_{\mathrm{h}} d\theta_{\mathrm{e}} d\theta_{\mathrm{h}} d\phi_{\mathrm{e}} d\phi_{\mathrm{h}}\ .$$

(S11)

Each element of the summations can be separated into a radial and an angular integral using eqs S3 and S4. The radial integral takes the form:

$$\int_{r_{\mathrm{e}}=0}^{r_{\mathrm{CdS}}} \int_{r_{\mathrm{h}}=0}^{r_{\mathrm{CdSe}}} R_{n_{\mathrm{e1}}, l_{\mathrm{e1}}} R_{n_{\mathrm{h1}}, l_{\mathrm{h1}}} \frac{\min(r_{\mathrm{e}}, r_{\mathrm{h}})^q}{\max(r_{\mathrm{e}}, r_{\mathrm{h}})^{q+1}} R_{n_{\mathrm{e2}}, l_{\mathrm{e2}}} R_{n_{\mathrm{h2}}, l_{\mathrm{h2}}} r_{\mathrm{e}}^2 r_{\mathrm{h}}^2 dr_{\mathrm{e}} dr_{\mathrm{h}}\ . \tag{S12}$$

This integral does not separate further and is evaluated numerically. The angular integral takes the form:

$$\int_{\theta_{\mathrm{e}}=0}^{\pi}\int_{\theta_{\mathrm{h}}=0}^{\pi}\int_{\phi_{\mathrm{e}}=0}^{2\pi}\int_{\phi_{\mathrm{h}}=0}^{2\pi} Y_{l_{\mathrm{e1}}}^{m_{\mathrm{e1}}\,*} Y_{l_{\mathrm{h1}}}^{m_{\mathrm{h1}}\,*} Y_q^p Y_q^{p\,*}\, Y_{l_{\mathrm{e2}}}^{m_{\mathrm{e2}}} Y_{l_{\mathrm{h2}}}^{m_{\mathrm{h2}}} \sin(\theta_{\mathrm{e}}) \sin(\theta_{\mathrm{h}})\, d\theta_{\mathrm{e}} d\theta_{\mathrm{h}} d\phi_{\mathrm{e}} d\phi_{\mathrm{h}}\ , \qquad \text{(S13)}$$

which can be separated into an electron and a hole contribution. Using the relationship $Y_l^{m^*} = (-1)^m Y_l^{-m}$ allows us to write the electron part as:

$$\int_{\theta_{\mathrm{e}}=0}^{\pi}\int_{\phi_{\mathrm{e}}=0}^{2\pi} (-1)^{m_{\mathrm{e1}}} Y_{l_{\mathrm{e1}}}^{-m_{\mathrm{e1}}} Y_q^p Y_{l_{\mathrm{e2}}}^{m_{\mathrm{e2}}}\, d\theta_{\mathrm{e}} d\phi_{\mathrm{e}}\ . \qquad \text{(S14)}$$

The solutions to angular integrals over the spherical harmonics can be expressed using the Wigner 3-j symbols, which gives:

$$\sqrt{\frac{(2l_{\mathrm{e1}}+1)(2q+1)(2l_{\mathrm{e2}}+1)}{4\pi}}\,(-1)^{m_{\mathrm{e1}}} \begin{pmatrix} l_{\mathrm{e1}} & q & l_{\mathrm{e2}} \\ 0 & 0 & 0 \end{pmatrix} \begin{pmatrix} l_{\mathrm{e1}} & q & l_{\mathrm{e2}} \\ -m_{\mathrm{e1}} & p & m_{\mathrm{e2}} \end{pmatrix}. \qquad \text{(S15)}$$

For the hole we obtain a similar expression; again we just substitute the index h for e. However, because here we consider the term $Y_q^{p^*}$, we also have to use $-p$ instead of p in the second Wigner 3-j symbol and pick up an additional factor of $(-1)^p$.

Because the operator for the homogeneous electric field acts on electron and hole wavefunctions separately, it can be calculated using the single-particle wavefunctions given by eq S4. For the electron, the effect is calculated as:

$$-eE_z\langle \psi^{\mathrm{e}}_{n_{\mathrm{e1}},l_{\mathrm{e1}},m_{\mathrm{e1}}} | z | \psi^{\mathrm{e}}_{n_{\mathrm{e2}},l_{\mathrm{e2}},m_{\mathrm{e2}}} \rangle\ . \qquad \text{(S16)}$$

This expression can again be simplified by separating the wavefunction into a radial and an angular part and expressing $z = r\cos(\theta) = 2r\sqrt{\frac{\pi}{3}}\,Y_1^0$ using spherical harmonics, which gives:

$$\langle \psi^{\mathrm{e}}_{n_{\mathrm{e1}},l_{\mathrm{e1}},m_{\mathrm{e1}}} | z | \psi^{\mathrm{e}}_{n_{\mathrm{e2}},l_{\mathrm{e2}},m_{\mathrm{e2}}} \rangle = \int_{r_{\mathrm{e}}=0}^{r_{\mathrm{CdS}}}\int_{\theta_{\mathrm{e}}=0}^{\pi}\int_{\phi_{\mathrm{e}}=0}^{2\pi} R_{n_{\mathrm{e1}},l_{\mathrm{e1}}} Y_{l_{\mathrm{e1}}}^{m_{\mathrm{e1}}\,*} r_{\mathrm{e}} 2\sqrt{\frac{\pi}{3}}\, Y_1^0 R_{n_{\mathrm{e2}},l_{\mathrm{e2}}} Y_{l_{\mathrm{e2}}}^{m_{\mathrm{e2}}} r_{\mathrm{e}}^2 \sin(\theta_{\mathrm{e}})\, dr_{\mathrm{e}} d\theta_{\mathrm{e}} d\phi_{\mathrm{e}}\ . \qquad \text{(S17)}$$

This can be split into radial and angular contributions. The radial part is given by:

$$\int_{r_{\mathrm{e}}=0}^{r_{\mathrm{CdS}}} R_{n_{\mathrm{e1}},l_{\mathrm{e1}}} R_{n_{\mathrm{e2}},l_{\mathrm{e2}}} r_{\mathrm{e}} r_{\mathrm{e}}^2\, dr_{\mathrm{e}}\ , \qquad \text{(S18)}$$

which is evaluated numerically. The angular integral can again be expressed using the Wigner 3-j symbols:

$$\sqrt{\frac{(2l_{\mathrm{e1}}+1)(2+1)(2l_{\mathrm{e2}}+1)}{4\pi}}\,(-1)^{m_{\mathrm{e1}}} \begin{pmatrix} l_{\mathrm{e1}} & 1 & l_{\mathrm{e2}} \\ 0 & 0 & 0 \end{pmatrix} \begin{pmatrix} l_{\mathrm{e1}} & 1 & l_{\mathrm{e2}} \\ -m_{\mathrm{e1}} & 0 & m_{\mathrm{e2}} \end{pmatrix}. \qquad \text{(S19)}$$

For the hole, we again substitute h for e and remove the minus sign in eq S16. The eigenstates of the Hamiltonian then give the exciton energy levels and the eigenvectors give their coefficients in eq S5.

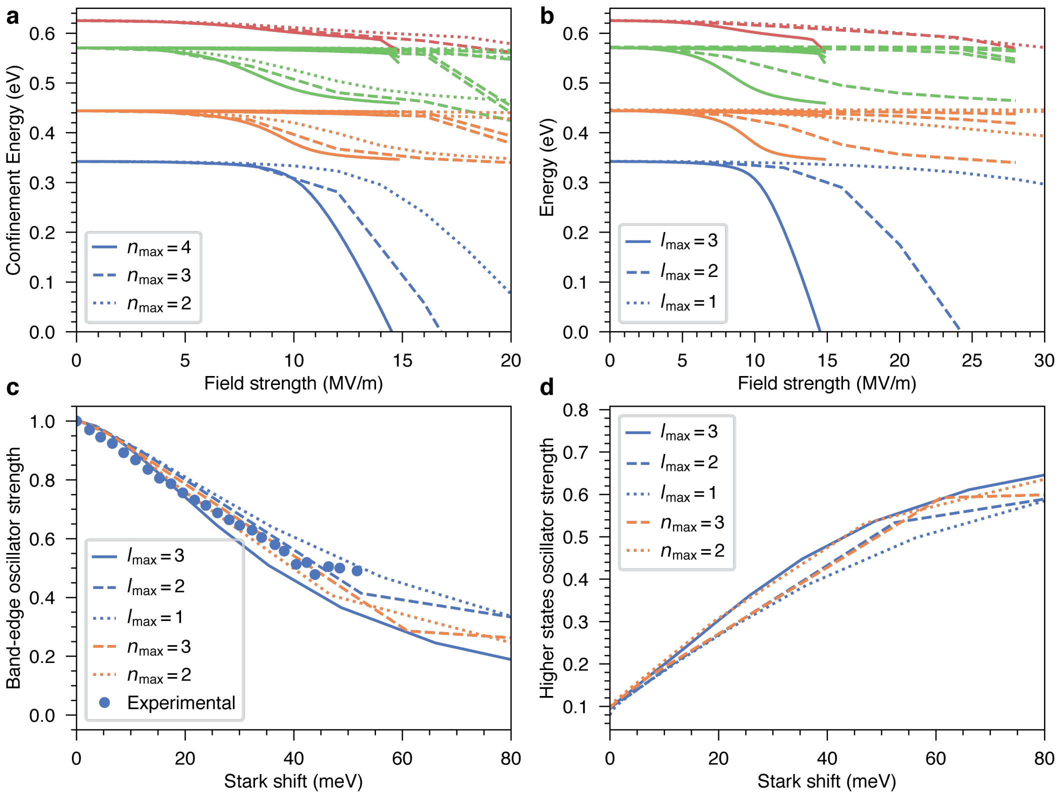

Figure S5. Analysis of the convergence of the calculations. (a) Field dependence of the exciton levels when limiting the highest quantum number *n*. (b) Same as panel a but for *l*. Even for the highest parameter values used, the calculations did not fully converge. (c) Loss of band-edge oscillator strength versus Stark shift for different bases. (d) Same as in panel c but for the oscillator strengths of the higher states.

Oscillator strengths in this work were calculated as the squared norm of the overlap of the electron and hole wavefunctions:[S10]

$$\left|\sum_{n_e,l_e,m_e,n_h,l_h,m_h} c_{n_e,l_e,m_e,n_h,l_h,m_h} \left\langle \psi^{(e)}_{n_e,l_e} \middle| \psi^{(h)}_{n_h,l_h} \right\rangle \right|^2. \tag{S20}$$

Section S8. Convergence of the Calculations

The eigenstates found in Figure S5 are only exact for a complete basis set, which would be infinitely big. We analyzed the convergence of the different properties by running calculations with different bases. Figure S5a shows the calculated excitonic energy levels as a function of applied field strength for different maximum quantum numbers n considered. The field necessary to cause a certain red-shift is highly dependent on the number of states considered, where, for a more exact calculation with higher $n_{\max}$, lower fields are required to cause a given shift. The same is true for the quantum number l, as shown in Figure S5b. For both numbers, calculations have not

converged as they are dependent on where the cut-off is chosen. Unfortunately, the computational cost quickly becomes prohibitive. However, while the exact dependence of the properties on the electric-field strength remains only approximative, we find that the dependence of derived quantities on one another converges much better. Figure S5c shows the oscillator strength of the band-edge transition against the Stark shift for different considered bases together with the experimental data. The spread between the different calculations is much smaller, indicating a better convergence. For the oscillator strength of the higher excited states, we make a similar observation, where all calculations line up reasonably well (Figure S5d). Thus, while the basis considered has an impact on the field needed to trigger a certain effect, the observables in our measurements are only weakly affected.

Section S9. Field Generated by an Elementary Charge

Neglecting the effect of surfaces, the potential due to a point charge located at the nanocrystal surface can be calculated by eqs S9 and S10. The $q = 1$-term gives rise to a homogeneous electric field and if the charge is placed along the z-axis at a distance r_0 from the nanocrystal center this term reads:

$$V_{\mathrm{C}}(r, \theta) = \frac{e}{4\pi\varepsilon\varepsilon_0} \cos(\theta) \frac{r}{r_0^2} \tag{S21}$$

Considering a charge positioned just outside of the nanocrystal at r_{ZnS}, the field is given by the gradient of eq S21, which for this specific case only has a z component. For $r_{\mathrm{ZnS}} = 4.5$ nm and $\varepsilon = 10$ this gives a homogeneous electric field strength of $\approx \frac{5\ \mathrm{MV}}{\mathrm{m}}$. This is in the right range to cause the effects observed experimentally. Note that our convergence analysis (Section S7) indicated that our calculations overestimate the necessary electric field to explain experimental observations. In addition, dielectric effects increase the field generated by a point charge,[S11] bringing the field due to a single point charge closer to the calculated value of 10 MV/m necessary to explain our findings.

References

(S1) Boyer, D.; Tamarat, P.; Maali, A.; Lounis, B.; Orrit, M. Photothermal Imaging of Nanometer-Sized Metal Particles Among Scatterers. *Science* **2002**, *297*, 1160–1163.

(S2) Gaiduk, A.; Yorulmaz, M.; Ruijgrok, P. V.; Orrit, M. Room-Temperature Detection of a Single Molecule's Absorption by Photothermal Contrast. *Science* **2010**, *330*, 353–356.

(S3) Chatterjee, R.; Pavlovetc, I. M.; Aleshire, K.; Kuno, M. Single Semiconductor Nanostructure Extinction Spectroscopy. *J. Phys. Chem. C* **2018**, *122*, 16443–16463.

(S4) Reiss, P.; Bleuse, J.; Pron, A. Highly Luminescent CdSe/ZnSe Core/Shell Nanocrystals of Low Size Dispersion. *Nano Lett.* **2002**, *2*, 781–784.

(S5) Boldt, K.; Kirkwood, N.; Beane, G. A.; Mulvaney, P. Synthesis of Highly Luminescent and Photo-Stable, Graded Shell CdSe/$Cd_xZn_{1-x}S$ Nanoparticles by In Situ Alloying. *Chem. Mater.* **2013**, *25*, 4731–4738.

(S6) le Feber, B.; Prins, F.; De Leo, E.; Rabouw, F. T.; Norris, D. J. Colloidal-Quantum-Dot Ring Lasers with Active Color Control. *Nano Lett.* **2018**, *18*, 1028–1034.

(S7) Galland, C.; Ghosh, Y.; Steinbrück, A.; Sykora, M.; Hollingsworth, J. A.; Klimov, V. I.; Htoon, H. Two Types of Luminescence Blinking Revealed by Spectroelectrochemistry of Single Quantum Dots. *Nature* **2011**, *479*, 203–207.

(S8) Hinterding, S. O. M.; Vonk, S. J. W.; van Harten, E. J.; Rabouw, F. T. Dynamics of Intermittent Delayed Emission in Single CdSe/CdS Quantum Dots. *J. Phys. Chem. Lett.* **2020**, *11*, 4755–4761.

(S9) Wu, S.; Xia, W. Exciton Polarizability and Absorption Spectra in CdSe/ZnS Nanocrystal Quantum Dots in Electric Fields. *J. Appl. Phys.* **2013**, *114*, 043709.

(S10) Klimov, V. I. *Nanocrystal Quantum Dots*; CRC Press, 2017.

(S11) Sercel, P. C.; Shabaev, A.; Efros, Al. L. Photoluminescence Enhancement through Symmetry Breaking Induced by Defects in Nanocrystals. *Nano Lett.* **2017**, *17*, 4820–4830.
